# Sonographic Brain Volume Growth Trajectories in VLBW and Clinical Determinants—Data from the NeoNEVS Project

**DOI:** 10.3390/children13020281

**Published:** 2026-02-18

**Authors:** Christian Brickmann, Renée Lampe, Irina Sidorenko, Nils Gauger, Julia Hauer, Marcus Krüger, Simon Loth

**Affiliations:** 1 School of Medicine and Health, Department of Pediatrics, Technical University of Munich, TUM University Hospital, 80804 Munich, Germany; 2 Clinic for Neonatology, Muenchen Klinik gGmbH, 81545 Munich, Germany; 3 Department of Clinical Medicine, Center for Digital Health and Technology, Orthopedic Department, Research Unit for Paediatric Neuroorthopedics and Cerebral Palsy of the Buhl-Strohmaier Foundation, Klinikum Rechts der Isar, School of Medicine and Health, Technical University of Munich, 81675 Munich, Germany

**Keywords:** VLBW, brain growth, ultrasound, brain volume, cerebral development

## Abstract

**Highlights:**

**What are the main findings?**

- Serial bedside cranial ultrasound with an ellipsoid model provides reproducible longitudinal estimates of total brain volume growth in VLBW infants during NICU stay.
- Longer duration of invasive mechanical ventilation is associated with slower ultrasound-derived brain volume growth.

**What are the implications of the main findings?**

- Internally derived percentile trajectories can help contextualize an individual infant’s brain growth pattern over time.
- This feasible bedside approach may support routine monitoring and hypothesis-driven risk stratification when interpreted alongside standard clinical and anthropometric data.

**Abstract:**

Background: Very Low Birth Weight preterm infants are at elevated risk for disrupted brain growth and later neurodevelopmental impairment. Bedside-accessible tools for monitoring cerebral development remain limited. Methods: In this retrospective pilot cohort study, 153 Very Low Birth Weight infants (<32 weeks gestational age and/or <1500 g) from two Level III Neonatal Intensive Care Units underwent serial cranial ultrasound assessments. Total brain volume was estimated using an ellipsoid formula derived from standardized imaging planes. Growth trajectories were analysed via linear mixed-effects modelling. Associations with clinical predictors—including invasive ventilation, sepsis, and somatic growth—were evaluated. Results: A total of 976 brain volume measurements were collected. Median cerebral volume increased from 164 cm^3^ to 275 cm^3^ across the hospital stay, corresponding to a median growth rate of 2.3 cm^3^/day (95% CI: 1.5–3.1). Duration of invasive mechanical ventilation was associated with reduced cerebral growth (*p* < 0.01, R^2^ = 0.26). Cerebral volume growth showed a weak but statistically significant correlation with head circumference percentile progression (*p* < 0.05, ρ = 0.16). Conclusions: Sonographic brain volumetry is a feasible and non-invasive method for tracking cerebral development in Very Low Birth Weight infants. These findings confirm significant associations between cerebral growth and head growth and identify prolonged invasive ventilation as a risk factor for impaired cerebral development.

## 1. Introduction

Preterm birth remains a major global health challenge and a leading contributor to neonatal morbidity and long-term neurodevelopmental impairment. Very Low Birth Weight infants, particularly those born at less than 32 weeks of gestation, are at elevated risk for disrupted brain growth during a critical period of rapid cerebral maturation, and impaired early brain growth has been linked to adverse cognitive, motor, and behavioral outcomes later in childhood [1]. This vulnerability reflects the interaction of immaturity with neonatal morbidities such as Bronchopulmonary Dysplasia (BPD), Intraventricular Hemorrhage (IVH), Necrotizing Enterocolitis (NEC), and sepsis, all of which have been associated with altered brain development and poorer long-term outcomes [2,3,4,5]. In addition, longer Neonatal Intensive Care Unit hospitalization and higher postmenstrual age at discharge—often reflecting cumulative illness burden—have been inversely associated with developmental outcomes [3,6,7,8]. Reliable, feasible approaches to monitor early brain growth trajectories are therefore needed.

Magnetic resonance imaging (MRI) is exceptionally well suited for neonatal neuroimaging because it provides high-resolution structural detail, quantitative volumetry, and sensitivity to subtle microstructural injury [9]. MRI is therefore considered the reference method for comprehensive brain assessment [10]. However, MRI is operationally demanding in preterm infants: it commonly requires elaborate preparation, transport outside the Neonatal Intensive Care Unit, and careful thermal and cardiorespiratory management; repeated assessments across admission are difficult to implement in routine care [11,12]. Consequently, cranial ultrasonography (cUS) remains the primary imaging modality in Neonatal Intensive Care Units worldwide due to its portability, safety, bedside applicability, and cost-effectiveness [13,14].

Beyond lesion detection, cUS can support quantitative monitoring. Several groups have shown that linear two-dimensional measurements can be combined using an ellipsoid approximation to estimate the total brain volume (TBV), providing a practical surrogate of whole-brain development with meaningful clinical correlates [15,16]. Prior studies demonstrated the feasibility of cUS-based volumetry and associations at term-equivalent age, and sequential cUS has been used to capture postnatal brain growth patterns and relate them to perinatal risk factors and later outcomes [14,17,18]. While targeted measurements of discrete structures (for example, corpus callosum or cerebellum) may also carry prognostic information [19], such measures can be more operator-dependent and less consistently obtainable during routine bedside imaging. To maximize reproducibility and scalability, our approach focuses on two standardized planes (midline sagittal and coronal) to derive three orthogonal axes for whole-brain TBV estimation, prioritizing a robust and repeatable bedside workflow. Recent standardized ultrasound-based scoring approaches further support structured cUS tools as complementary bedside methods [13].

Nevertheless, longitudinal full-brain volumetric data in Very Low Birth Weight infants using serial ultrasound remain scarce, and percentile curves for postnatal TBV trajectories assessed by ultrasound are not yet established [13,20].

Accordingly, this pilot study aimed to assess the feasibility of longitudinal cUS-derived TBV tracking during NICU stay, derive internal TBV trajectories/percentiles for descriptive context, and explore associations with clinical factors; we hypothesized that ultrasound-derived total brain volume growth follows a measurable trajectory and is attenuated in infants with greater morbidity.

## 2. Methods

### 2.1. Study Design and Population

We conducted a retrospective, two-centre pilot cohort study of consecutively treated preterm infants admitted to two Level-III neonatal units in Germany (January 2019–December 2021). The pilot study design aimed to assess feasibility and derive internal trajectories of total brain volume (TBV) during hospitalization; secondary analyses evaluated associations between TBV growth and clinically relevant neonatal morbidities and exposures.

**Figure 1:**
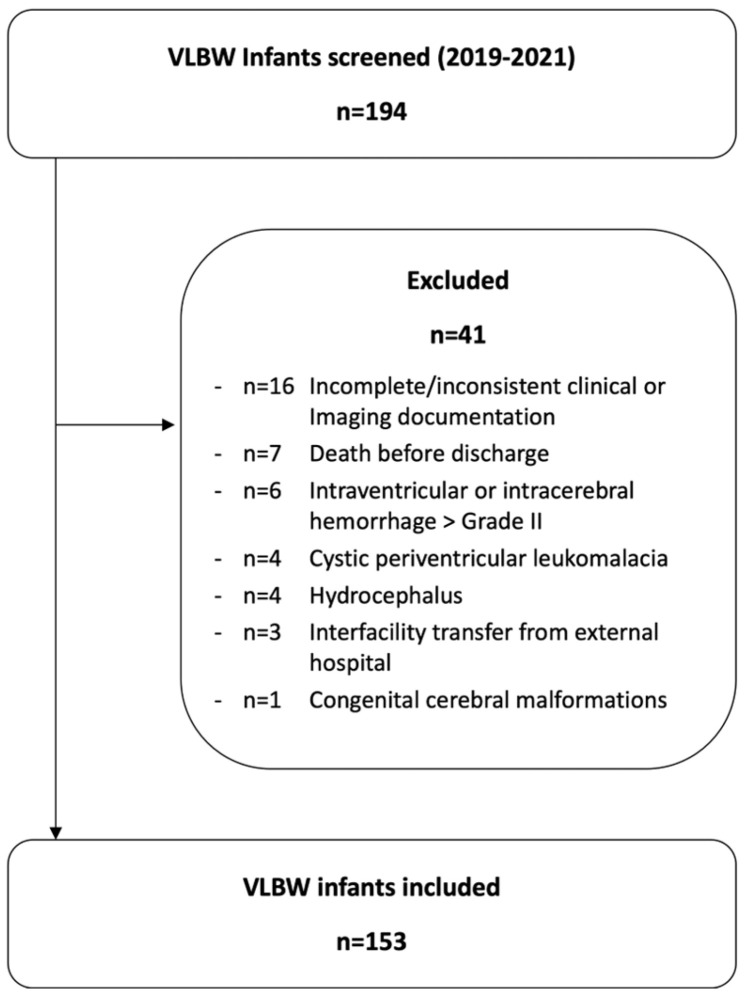
Flow chart of the included patients.

Eligibility criteria were birth at <32 weeks gestational age and birth weight <1500 g. Of 194 infants screened, 41 were excluded to minimize bias in ultrasound-based TBV estimation and to ensure adequate longitudinal data completeness. Exclusion criteria were intraventricular or intracerebral hemorrhage >Papile grade II, cystic periventricular leukomalacia, hydrocephalus, congenital cerebral malformations, death before discharge, interfacility transfer (outborn status), and incomplete or inconsistent clinical or imaging documentation (Figure 1). These exclusions were applied to avoid distortion of TBV estimates by major destructive pathology and to enable longitudinal modelling; consequently, generalizability is limited to VLBW infants without major structural brain injury.

### 2.2. Techniques

Routine cranial ultrasonography was performed by neonatologists at both sites using the same system (Philips Affinity 70; Philips C 8–5 MHz broadband microconvex transducer, Bothell, WA, USA). Examinations followed institutional schedules consistent with national recommendations (Days of Life 1, 3, 7, 14, and 28; thereafter every 14 days until discharge), with additional scans as clinically indicated. Image acquisition adhered to German Society for Ultrasound in Medicine guidance using predefined coronal and sagittal planes with standardized documentation.

Stored images were re-evaluated offline in the institutional PACS (Centricity Universal Viewer Zero Footprint, GE Healthcare). Linear brain dimensions were measured directly on archived frames; measurements were obtained independently by neonatologists trained in neonatal neurosonography. TBV was calculated using an ellipsoid approximation based on three orthogonal parenchymal axes—coronal biparietal diameter (BiPD), sagittal anteroposterior axis (APA), and sagittal vertical axis (VA):

TBV = (4/3) × π × (BiPD/2) × (APA/2) × (VA/2).

**Figure 2:**
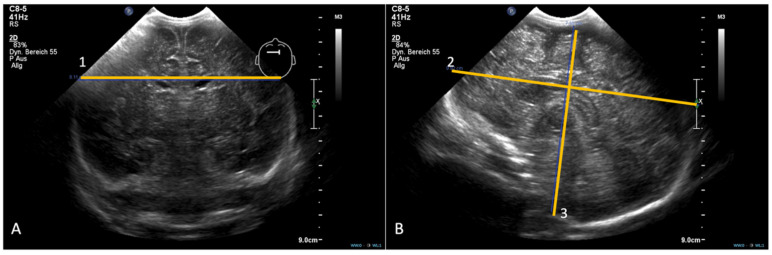
Examples of the coronal (**A**) and sagittal planes (**B**), including the biparietal diameter (1), anterioposterior axis (2) and vertical axis (3).

Distances were recorded in millimetres and TBV was reported in cubic centimetres. BiPD was defined as the maximal lateral distance between the outer parietal cortices in the third coronal plane. In the midline sagittal plane, APA was measured as the fronto-occipital length along a horizontal reference line through the corpus callosum; VA was the maximal craniocaudal distance from the cortical surface beneath the transducer to the foramen magnum, oriented perpendicular to the brainstem and defined using characteristic echogenic landmarks (Figure 2).

The ellipsoid approach was chosen to enable reproducible serial bedside volumetry during NICU admission. MRI is the reference standard for neonatal volumetry but is not feasible for frequent longitudinal assessment in routine care. Detailed compartmental volumetry, including intraventricular and extracerebral cerebrospinal fluid spaces, requires reliable delineation of fluid–parenchyma boundaries and is technically demanding, even in MRI-based workflows; ventricular dimensions and ventricular volume were therefore not quantified. Measurement lines were placed at the outer contour of the brain parenchyma rather than at the cranial bone reflection to minimize inclusion of extracerebral spaces. Regional measures were not pursued because they are more operator-dependent and were not consistently available retrospectively.

Measurement reproducibility was assessed using Intraclass Correlation Coefficients (ICC) for BiPD, APA, VA, and derived TBV. Intra-observer repeatability was evaluated using a two-way mixed-effects, absolute-agreement, single-measures model [ICC(3,1)], and inter-observer agreement using a two-way random-effects, absolute-agreement, single-measures model [ICC(2,1)], each with 95% confidence intervals. Reliability analyses were based on 82 paired observations from 15 additional infants, with independent review by two neonatologists with expertise in neonatal neurosonography.

### 2.3. Data Measurements

Clinical data were abstracted from the medical record and included gestational age and body composition parameters at birth and discharge, neonatal morbidities (PDA, BPD, sepsis, NEC or focal intestinal perforation, IVH grade I–II), and respiratory support variables (occurrence and duration of invasive and non-invasive mechanical ventilation).

### 2.4. Statistical Analysis

Analyses were performed in SPSS Statistics v31 (IBM Corp., Walldorf, Germany) and GraphPad Prism v10 (GraphPad Software). Two-sided *p* < 0.05 was considered significant. We use the term “correlation” only for Spearman rank correlations (reported as ρ), and the term “association” for regression-based relationships estimated in the mixed-effects model (reported as fixed-effect estimates with 95% confidence intervals and *p*-values); R^2^ values are reported as marginal/conditional R^2^ for mixed models.

*Longitudinal modelling (primary analysis).* Repeated TBV measurements were analysed using a Linear Mixed-effects Model (restricted maximum likelihood; Satterthwaite degrees of freedom). Fixed effects included Day of Life (continuous), gestational age at birth (continuous), and their interaction. No fixed intercept was specified, so coefficients represent period-specific growth terms. Random effects comprised subject-specific intercepts and slopes for Day of Life and gestational age with an unstructured covariance matrix. Within-infant serial correlation was modelled using a repeated Day of Life term with diagonal covariance at the subject level. Model adequacy was evaluated by information criteria (−2RLL, AIC, AICC, BIC), residual inspection, and comparison of alternative covariance structures. Fixed-effect and subject-level predictions were exported for descriptive summaries and percentile estimation.

*Percentile derivation (internal framework).* Model-predicted TBV values were grouped by Day of Life into 5-day bins (165–285 days). Within each bin, empirical percentiles (5th, 10th, 25th, 50th, 75th, 90th, 95th) with 95% confidence intervals were computed in SPSS using the weighted-average method. For figures, percentile trajectories were smoothed in GraphPad Prism using a smoothing spline; smoothing was used for visualization only, whereas reported percentiles and summary estimates were based on unsmoothed per-bin values.

*Comparative and association analyses.* Birth-to-discharge changes in ordinal/continuous variables were assessed using Wilcoxon signed-rank tests; categorical variables were analysed using Fisher’s exact test. Associations between TBV growth and clinical covariates were quantified using Spearman rank correlation (ρ) with exact *p*-values and 95% confidence intervals, while regression models are reported with 95% confidence intervals and R^2^ where applicable. For multivariable models, fixed-effect estimates with 95% confidence intervals, marginal and conditional R^2^, and influence diagnostics (Cook’s distance) are reported. Analyses used listwise deletion. Given the limited number of prespecified hypotheses, no multiplicity adjustment was applied. Sensitivity analyses refit the mixed model after excluding infants with <3 scans and, separately, modelled Day of Life using a restricted cubic spline.

### 2.5. Ethics Approval and Consent

The study complied with institutional policies and the Declaration of Helsinki. Owing to its retrospective design and anonymized data, consent for data use was covered by consent to treatment. The protocol was approved by the Ethics Committee of the Technical University of Munich (2024-460-S-KK). No clinical trial registration was required.

## 3. Results

Demographic characteristics of the study population are summarized in Table 1 and Table 2. A total of 153 preterm neonates were included, with a total of 976 cerebral volume measurements analysed over the course of their NICU admission.

**Figure 3:**
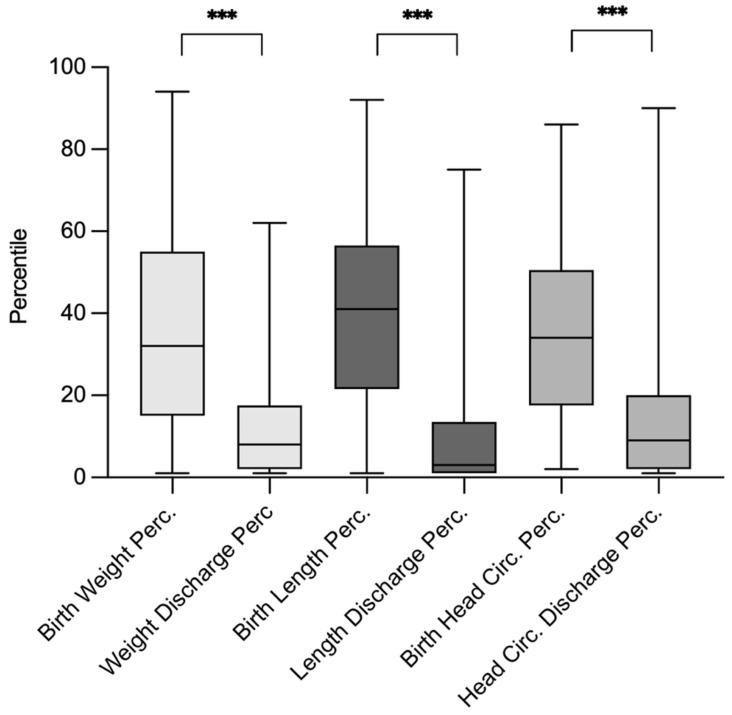
Body composition in percentile numbers at birth and at discharge. Abr.: Perc. = Percentile., *** = *p* < 0.001.

Clinical comparisons between birth and discharge showed significant declines in percentile rankings for weight (from a mean of 32 to 8, *p* < 0.01), length (from 41 to 3, *p* < 0.01), and head circumference (from 34 to 9, *p* < 0.01), consistent with postnatal growth restriction commonly observed in VLBW infants. Body composition of weight, length and head circumference in percentile numbers at birth and at discharge are displayed in Figure 3.

**Figure 4:**
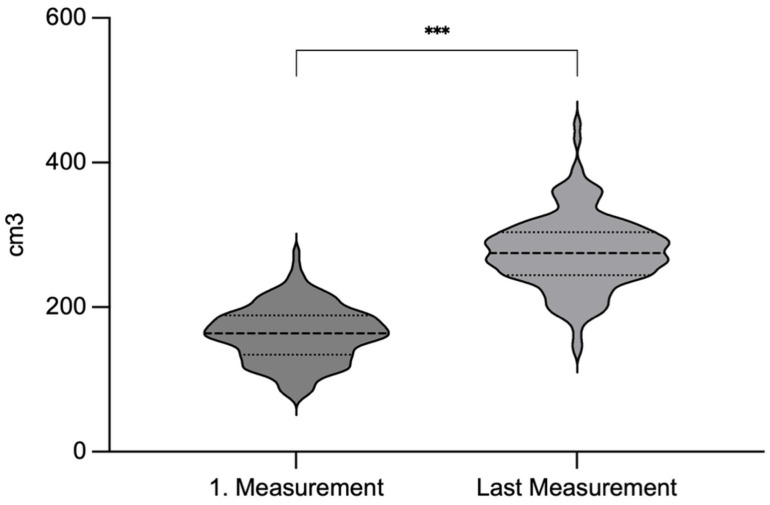
Comparison of brain volume between the first and the final measurement. Dotted lines indicate 25. and 75. percentile; dashed lines indicate 50. percentile. Abr.: *** = *p* < 0.001.

**Figure 5:**
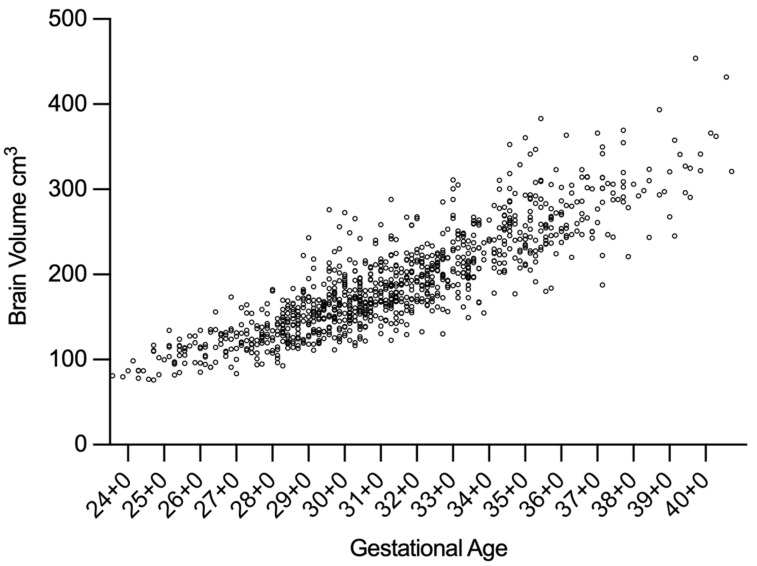
Scatterplot of the longitudinal brain volume measurements of all patients. Circles indicate brain volume measurement at the according corrected gestational age. Abr.: GA = Gestational Age.

**Figure 6:**
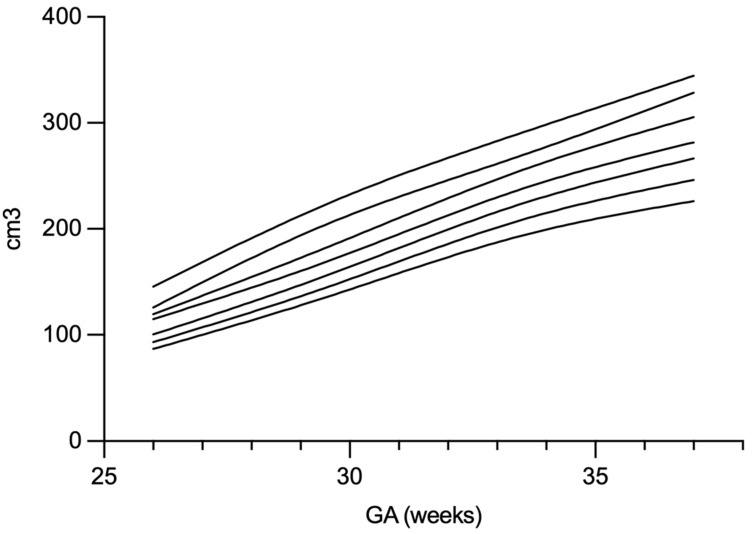
Lines show the percentiles of brain volume, including 5, 10, 25, 50, 75, 90, 95th percentile (26 + 3–37 + 1 weeks GA). Abr.: GA = Gestational Age.

The median brain volume at the time of the first measurement (usually Day of Life 1–3) was 164 cm^3^ (range: 77–276 cm^3^), increasing to 275 cm^3^ (range: 140–454 cm^3^) at the final measurement (see Figure 4). The overall median rate of cerebral volume growth was 2.3 cm^3^/day (95% CI: 1.5–3.1), as estimated using the linear mixed-effects model. Longitudinal brain volume data for all patients are illustrated in Figure 5. Percentile-based trajectories of brain volume growth are visualized in Figure 6.

A multiple linear regression analysis identified duration of invasive mechanical ventilation as a significant predictor of impaired brain volume growth (R^2^ = 0.26, *p* < 0.01). No other predictors showed a statistically significant association with brain volume growth in the regression model (Table 3). Additionally, a minor, but still significant correlation was observed between individual brain volume growth and the change in head circumference percentile from birth to discharge (ρ = 0.16, *p* < 0.05, 95% CI: −0.0021 to 0.32).

We observed excellent repeatability and agreement across all ultrasound measurements. Intra-observer reliability (n = 15 patients; 82 paired examinations; 328 parameter-level comparisons [82 examinations × 4 measures: coronal, sagittal-horizontal, sagittal-vertical, and TBV]) yielded ICCs of 0.943–0.980 across coronal, sagittal-horizontal, sagittal-vertical, and TBV, with minimal session drift. Inter-observer reliability was likewise high, with ICCs of 0.917–0.981 for linear dimensions and 0.975–0.981 for TBV across both reading sessions (Table 4).

## 4. Discussion

This pilot study shows that serial cranial ultrasound–based TBV estimation using an ellipsoid model is feasible for longitudinal bedside monitoring in VLBW preterm infants during routine NICU care. Median TBV increased from 164 cm^3^ to 275 cm^3^ across admission, corresponding to a median growth rate of 2.3 cm^3^/day, which is consistent with prior MRI- and ultrasound-derived estimates and supports the biological plausibility of this simplified approach [15,16,21]. While MRI remains the reference standard for neonatal volumetry and tissue-level characterization, this ellipsoid-based approach provides a simplified whole-brain estimate suitable for longitudinal bedside tracking rather than detailed anatomic segmentation. Using three orthogonal parenchymal dimensions from standardized coronal and midline sagittal planes, the protocol targets whole-brain development and supports scalable implementation.

*Main finding and clinical interpretation:* Duration of invasive mechanical ventilation (IMV) showed the strongest inverse association with TBV growth among examined variables. This is consistent with the literature suggesting that IMV may reflect illness severity and may also contribute to impaired brain development through hyperoxia/hypocapnia exposure and inflammation-related pathways [2,22]. Proposed mechanisms include altered cerebral perfusion, delayed myelination, and white-matter vulnerability during rapid postnatal growth. However, this association does not establish causality and should be interpreted cautiously, given potential residual confounding and the possibility that IMV is a marker of overall illness burden. The direction of effect is nonetheless congruent with reports linking longer mechanical ventilation to increased risk of neurodevelopmental impairment at two years, even after adjustment for perinatal covariates [23,24,25]. Because several morbidities (BPD, NEC, ROP, and sepsis) occurred in relatively small and overlapping subgroups, we did not perform a fully adjusted multivariable model across all morbidity categories.

*Internal TBV percentiles and relationship to head growth:* Internally derived TBV percentile curves were created to contextualize an individual infant’s serial trajectory relative to peers within this cohort. These curves are intended as descriptive, hypothesis-generating references to support interpretation of sustained low trajectories or downward crossing over time; without external validation, they should not be used as generalizable cutoffs or for decision-making in isolation [1,18]. Head circumference percentiles declined from birth to discharge and showed a modest but statistically significant Spearman correlation with TBV growth, supporting the concept that head circumference is an imperfect surrogate for parenchymal growth but may provide complementary, low-burden information for risk stratification [26,27,28]. These findings fit with clinical practice where bedside anthropometrics remain essential, but direct volumetric monitoring may offer earlier responsiveness.

*Somatic growth context:* The observed decline in anthropometric percentiles (weight, length, head circumference) underscores persistent extrauterine growth restriction in VLBW infants, which has been associated with adverse neurodevelopment, whereas head-sparing growth may be protective [2]. Given the ongoing debate about whether preterm infants should match in utero growth velocities, guidance increasingly supports combining reference curves with individualized longitudinal trajectories [29,30]. TBV trajectories may complement this framework by providing a direct measure of cerebral growth alongside somatic indices.

*Methodological considerations and feasibility:* Percentiles were derived from model-predicted TBV grouped into 5-day bins, with smoothing used only for visualization; curve tails require cautious interpretation where observations are sparse. The mixed-effects framework accounted for repeated measures and heterogeneity, and sensitivity analyses (excluding infants with <3 scans; alternative Day of Life specification) yielded consistent estimates, supporting robustness of the growth rate and IMV association. Our results also reinforce the expanding role of cUS beyond lesion detection: standardized acquisition and modern equipment enable quantitative whole-brain growth assessment at the bedside that aligns with MRI benchmarks without transport or sedation, and reliability was excellent for linear dimensions and derived TBV [13,15,16,21].

*Limitations and generalizability:* First, percentile curves are internally derived and not externally validated; they are descriptive rather than normative clinical standards. Second, the retrospective design introduces potential selection and measurement bias; observed relationships are associative and may reflect residual confounding. Although key covariates were considered, unmeasured confounders—including illness severity, nutrition, and hemodynamic instability—may partly explain associations. Systemic inflammation was approximated by clinical diagnoses (e.g., sepsis, NEC) rather than time-dependent inflammatory markers (e.g., serial C-reactive protein, leukocytes, cytokine profiles) or other physiologic indicators, which may co-occur with respiratory instability and independently affect cerebral development [31,32]. Third, the ellipsoid model does not capture regional maturation, focal white-matter abnormalities, ventricular contributions to intracranial size, or shape-related changes that are better resolved by MRI; regional structures (e.g., cerebellum, corpus callosum) can carry prognostic information in prematurity, and altered maturation in these regions has been linked to later outcomes. These measures were not pursued because the primary aim was a scalable bedside tool using routinely obtainable planes; robust cerebellar or callosal quantification often requires additional views and is more operator-dependent [19,22,33]. Fourth, ventricular dimensions were not quantified, so ventricular contributions to TBV and ventricular-corrected parenchymal volume cannot be assessed; this was a deliberate feasibility decision given the technical demands of compartmental volumetry. Additionally, excluding infants with Grade > II IVH, cystic periventricular leukomalacia, hydrocephalus, and congenital malformations limits generalizability and may bias the cohort toward healthier VLBW infants; in higher-risk infants, structural distortion and ventricular enlargement may require alternative metrics. Our measurement lines targeted the outer parenchymal contour rather than the cranial bone reflection to minimize extracerebral space inclusion. Finally, detailed nutritional exposure was not available in standardized form across centres and could not be incorporated; nutrition-related residual confounding may influence both somatic growth and TBV trajectories [14,15,34,35]

*Future directions:* Ongoing follow-up will link TBV trajectories to standardized neurodevelopmental assessments, consistent with evidence that early brain growth relates to later cognitive and motor outcomes [1,17,36]. Future work should integrate regional measures and Doppler indices, add standardized ventricular metrics, and incorporate systematically collected exposures—particularly nutrition—within larger datasets to enable more comprehensive multivariable modelling. Subsequent steps will also apply multivariable and machine-learning approaches to clarify the joint impact of neonatal morbidities on brain growth and longer-term outcomes.

## 5. Conclusions

This pilot study shows that total brain volume in VLBW preterm infants can be quantified reliably from routine cranial ultrasound using an ellipsoid model. Growth trajectories were clinically meaningful and most sensitive to the duration of invasive ventilation. Internally derived percentile curves may help contextualize longitudinal TBV growth during hospitalization and support hypothesis generation regarding atypical trajectories. Sonographic brain volume tracking could complement routine monitoring and, where available, term-equivalent MRI, but clinical thresholds and prognostic performance require external validation. Embedding sonographic brain volume tracking into routine NICU care may refine monitoring of cerebral growth and help identify infants who may benefit from closer follow-up or additional diagnostic evaluation. Prospective validation with long-term outcomes is needed.

## Figures and Tables

**Table 1:** Demographic cohort data. Abr.: GA = Gestational Age; IQR = Interquartile Range; Perc. = Percentile; UA = Umbilical Artery; IMV = Invasive Mechanical Ventilation; NIV = Non-Invasive Ventilation.

Demographic Data n = 153	Birth		Discharge	
	Median	IQR 25/75	Median	IQR 25/75
GA (week + day)	28 + 6	27 + 2/29 + 6		
Weight (g)	1070	880/1260	2390	2130/2640
Weight Perc.	32	15/55	8	2/18
Length (cm)	37	35/39	45	44/47
Length Perc.	41	22/57	3	1/14
Head circumference (cm)	26	25/28	32	31/33
Head circumference Perc.	34	18/51	9	2/20
APGAR 1	6	5/7		
APGAR 5	8	7/9		
APGAR 10	9	8/9		
pH UA	7.3	7.3/7.4		
IMV (h)	0	0/72		
NIV (h)	696	372/1164		

**Table 2:** Morbidity cohort data. Abr.: IMV = Invasive Mechanical Ventilation; NIV = Non-Invasive Ventilation; PDA = Patent Ductus Arteriosus; BPD = Bronchopulmonary Dysplasia; AIS = Amnion Infection Syndrome; EOS = Early Onset Sepsis; LOS = Late Onset Sepsis; BC = Blood Culture; NEC = Necrotizing Enterocolitis; FIP = Focal Intestinal Perforation; IVH = Intraventricular Hemorrhage.

Morbidity Data	Overall Number	%	Number in Category	%
Sex (male/female)	68/85	44/56		
Birth Mode (spontaneous/Cesarean Section)	8/145	5/95		
IMV (Yes/No)	63/90	41/59		
NIV (Yes/No)	153/0	100/0		
PDA	35	23		
None			118	77
Medical Treatment			34	22
Surgical Treatment			1	1
BPD	19	12		
None			134	88
Mild			12	8
Moderate			3	2
Severe			4	2
Sepsis	55	36		
None			98	64
AIS			13	9
EOS BC negative			2	1
EOS BC positive			3	1
LOS BC negative			16	11
LOS BC positive			21	14
NEC/FIP	10	7		
None			143	93
Medical Treatment			2	1
Surgical Treatment			8	6
IVH	17	11		
None			136	89
I°			14	9
II°			3	2

**Table 3:** Results of multiple regression analysis of brain growth with body composition at birth and at discharge and clinical parameters. Abr.: *** = *p* < 0.01; GA = Gestational Age; Perc. = Percentile; pH UA = Umbilical Artery pH; NIV = Non-Invasive Ventilation; h = hours.

Multiple Regression (R^2^: 0.26) of Brain Growth to:	*p*-Value	95% CI
GA	0.14	0.40–8.4
Birth Weight Perc.	0.27	−0.0017–0.012
Birth Length Perc.	0.2	−0.0068–0.002
Birth Head Circumference Perc.	0.16	−0.0014–0.007
Weight Discharge Perc	0.32	−0.0060–0.001
Length Discharge Perc.	0.36	−0.0033–0.01
Head Circumference Discharge Perc.	0.15	−0.0029–0.008
APGAR ′1	0.34	−0.0012–0.008
APGAR ′5	0.17	−0.069–0.024
APGAR ′10	0.72	−0.025–0.15
pH UA	0.08	−0.13–0.09
Duration of Mechanical Ventilation	***	−0.95–0.05
NIV	0.46	−0.00059–−9.1 × 10^5^
NIV (h)	0.33	−0.40–0.89

**Table 4:** Intra- and interrater reliability measurements of 15 patients covering the three sonographic planes and the resulting TBV. Abr.: ICC = Interclass Correlation Coefficient; CI = Confidence Interval; TBV = Total Brain Volume.

Measurements	Interrater ICC (CI 95)	Intrarater ICC (CI 95)
Biparietal Coronal	0.965 (0.939–0.979)	0.975 (0.961–0.984)
Sagittal anterio-posterior	0.971 (0.955–0.981)	0.978 (0.965–0.986)
Sagittal Vertical	0.935 (0.870–0.964)	0.943 (0.911–0.963)
TBV	0.975 (0.938–0.987)	0.980 (0.967–0.988)

## Data Availability

Clinical data were recorded and stored in pseudonymized form within the study-site network for 10 years (§630f BGB). No data are shared with third parties; access is restricted to the study team via the hospital network and a password-protected database. Public reporting uses anonymized data only. De-identified datasets generated and/or analysed during the study are available from the corresponding author on reasonable request.
